# Effect of Direct Versus Indirect Bonding Techniques on Fixed Retainer Failure Rates: A Systematic Review and Meta-Analysis

**DOI:** 10.7759/cureus.102821

**Published:** 2026-02-02

**Authors:** Mohamed A Marei, Lujain E Failakawi, Fatemah Y Alhouti, Mohammed Y Alshamly, Omar Yousry Mostafa

**Affiliations:** 1 Orthodontics, Ministry of Health, Alexandria, EGY; 2 Oral and Maxillofacial Surgery, Ministry of Health, Kuwait, KWT; 3 Orthodontics, Cairo University, Cairo, EGY

**Keywords:** bonding, failure rate, fixed lingual bonded retainer, fixed retainer, indirect bonding

## Abstract

Bonded fixed orthodontic retainers frequently fail, yet it remains uncertain whether laboratory-based indirect bonding, including computer-aided design and computer-aided manufacturing (CAD/CAM)-assisted techniques, reduces failure rates compared with conventional chair-side direct bonding. Following Preferred Reporting Items for Systematic Reviews and Meta-Analyses (PRISMA) guidelines, PubMed, Scopus, Cumulative Index to Nursing and Allied Health Literature (CINAHL), Cochrane Library, Dentistry & Oral Science Source, Google Scholar, ClinicalTrials.gov, and other trial registers were searched from inception to November 2025. Randomized controlled trials (RCTs) and prospective or retrospective cohort studies comparing indirect with direct bonding and reporting retainer failure were eligible. Two reviewers independently screened studies, extracted data, and assessed risk of bias using the Revised Cochrane Risk of Bias tool for randomized trials (RoB 2) for randomized trials and the Newcastle-Ottawa Scale (NOS) for cohort studies. Hazard ratios (HRs) for first failure were pooled using a random-effects model, and heterogeneity was quantified with the I² statistic. The protocol was registered in the International Prospective Register of Systematic Reviews (PROSPERO; registration number: CRD420251003540). Fifteen studies, including 13 RCTs and two cohort studies involving 1,481 patients, were included. The pooled analysis for mandibular retainers demonstrated a significantly higher failure risk in the direct bonding group (HR = 1.41, 95% CI 1.12-1.79, p = 0.004), with low heterogeneity (I² = 14%). For maxillary retainers, no statistically significant difference was observed between bonding techniques (HR = 1.28, 95% CI 0.90-1.84, p = 0.17). Overall risk of bias was low to moderate, with inadequate blinding being the most common limitation. Updated evidence indicates a significantly higher failure risk for directly bonded mandibular retainers compared with indirect bonding, while outcomes for maxillary retainers appear comparable between techniques. Indirect bonding may therefore offer improved mandibular retainer survival, although further well-controlled trials are required to confirm this effect.

## Introduction and background

Maintaining the results of orthodontic treatment poses a longstanding clinical challenge due to the natural tendency of teeth to relapse. Relapse is largely attributed to persistent periodontal fiber tension and muscular imbalance, which persist even after active treatment [1]. To counteract these forces and stabilize the dentition, various types of retainers have been developed, with fixed retainers, particularly the mandibular 3-3 bonded retainer, being among the most commonly employed due to their effectiveness and low reliance on patient compliance [2]. Traditionally, the direct bonding technique has been the preferred method for placing fixed retainers. This approach involves chairside placement of the retainer wire and composite application directly onto the teeth. In contrast, the indirect bonding technique involves laboratory-based preparation where the wire and composite pads are first positioned on a cast before being transferred to the patient’s mouth via a transfer tray. Advocates of the indirect method suggest it offers greater precision, shorter chairside time, and improved isolation, though its impact on clinical failure rates remains uncertain [3]. Despite the widespread use of both techniques, bond failure remains a frequent complication, often necessitating retreatment and raising concerns about long-term stability. While some studies suggest improved outcomes with indirect bonding due to controlled placement and enhanced moisture control, others report similar or higher failure rates compared to direct techniques [4]. These inconsistencies have created ambiguity regarding the optimal bonding approach.

In addition to technique-related factors, the bonding protocol itself may influence retainer survival. Variations in enamel preparation, etching concentration and duration, primer composition, and adhesive systems have been shown to affect bonding performance. Differences in enamel conditioning and adhesive systems have been shown to affect rebonding success and may therefore influence retainer survival. Laboratory-based indirect bonding, including computer-aided design and computer-aided manufacturing (CAD/CAM)-assisted workflows, may allow better control over composite placement and moisture isolation, which could partly explain differences in reported failure rates. Although previous systematic reviews have compared the effectiveness of different retainer designs and materials, few have focused specifically on failure rates associated with bonding techniques across both arches, including newer CAD/CAM-based systems. Therefore, the present systematic review and meta-analysis aimed to evaluate the failure rates of mandibular and maxillary fixed retainers placed using direct versus indirect bonding techniques. By synthesizing evidence from clinical studies, this review aims to clarify whether one technique demonstrates superior clinical performance and to provide evidence-based guidance for orthodontic retention protocols [5]. Unlike previous reviews, this study incorporates time-to-event analysis and includes contemporary CAD/CAM retainers.

## Review

Methods

Protocol and Registration

This meta-analysis was registered on the International Prospective Register of Systematic Reviews (PROSPERO; registration number: CRD420251003540). This systematic review was conducted in accordance with the Preferred Reporting Items for Systematic Reviews and Meta-Analyses (PRISMA) guidelines [6] to ensure that the review was done in a clear and organized way. We also updated the PROSPERO record to match the final review process.

Eligibility Criteria and Review Question 

The research question was formulated using the Population, Intervention, Comparator, Outcome (PICO) framework [7]. The population included patients who had completed orthodontic treatment and received a fixed retainer. The intervention was the indirect bonding technique for placement of the retainer; the comparator was the direct bonding technique. The outcome assessed was the failure rate of the fixed retainer. In this Review, we included both randomized clinical trials, randomized controlled trials (RCTs) and cohort studies.

Information Sources and Search Strategy

An electronic database search was done, including PubMed, Scopus, Cumulative Index to Nursing and Allied Health Literature (CINAHL) Plus (EBSCO), Cochrane Library, Dentistry & Oral Science Source, and the Trials Meta Register, up to November 2025. The search strategy used a combination of MeSH terms and Boolean operators so that relevant studies could be obtained. Also, a manual search was performed using Google Scholar and ClinicalTrials.gov, applying the same MeSH-based strategy. The full electronic search strategy is provided in Appendix A.

Study Selection Process

RCTs and prospective or retrospective cohort studies that reported the failure rates as outcomes were included. All search results were imported into the Rayyan software (Qatar Computing Research Institute (QCRI), Hamad Bin Khalifa University, Doha, Qatar) for screening. The study selection process was done in two phases by two independent reviewers (MM and MA). First, titles and abstracts were screened to exclude irrelevant studies. Then, the full texts of studies were reviewed for final inclusion. The final included studies were imported into Zotero (Corporation for Digital Scholarship, Vienna, VA) for citation.

Data Collection Process

Study screening and data extraction were performed independently by two reviewers, with disagreements resolved by consensus. Inter-rater agreement was assessed and is now reported to enhance transparency. Data extraction included the following items: author, year, study design, sample size, follow-up duration, etchant/adhesive used, type of retainer wire, failure rate results, and corresponding p-values and hazard ratios (HRs), reported or calculated when not provided directly. The extracted data were checked more than once for accuracy, and any discrepancies were resolved by re-reviewing the original studies.

Risk of Bias Assessment

The Cochrane Collaboration’s tool [8] was used by two authors to assess the risk of bias in the included studies (RCTs). This tool covers several key domains. After answering the questions for each domain, each study was rated as having a low, unclear, or high risk of bias. The Newcastle-Ottawa Scale (NOS) [9] was used for the cohort studies.

Statistical Analysis

In this meta-analysis, the retainer failure was assessed using failure rates and HRs. For each included study, we extracted the reported HR and its 95% confidence interval (CI) whenever available. When HRs were not reported, they were approximated from binary outcome data using established methods; these estimates assume proportional hazards and should be interpreted cautiously. This approach has been previously used in similar orthodontic and implant survival meta-analyses, as in the study by Tierney et al. [10]. 

Heterogeneity among studies was assessed using the I² statistic to evaluate differences in effect estimates. The meta-analysis was conducted for studies with available quantitative data using Review Manager (RevMan) version 5.3 (The Cochrane Collaboration, 2020, London, UK). Due to variations in follow-up durations across the included studies, a random-effects model was applied to account for potential heterogeneity.

Results

Study Selection and Characteristics

**Figure 1:**
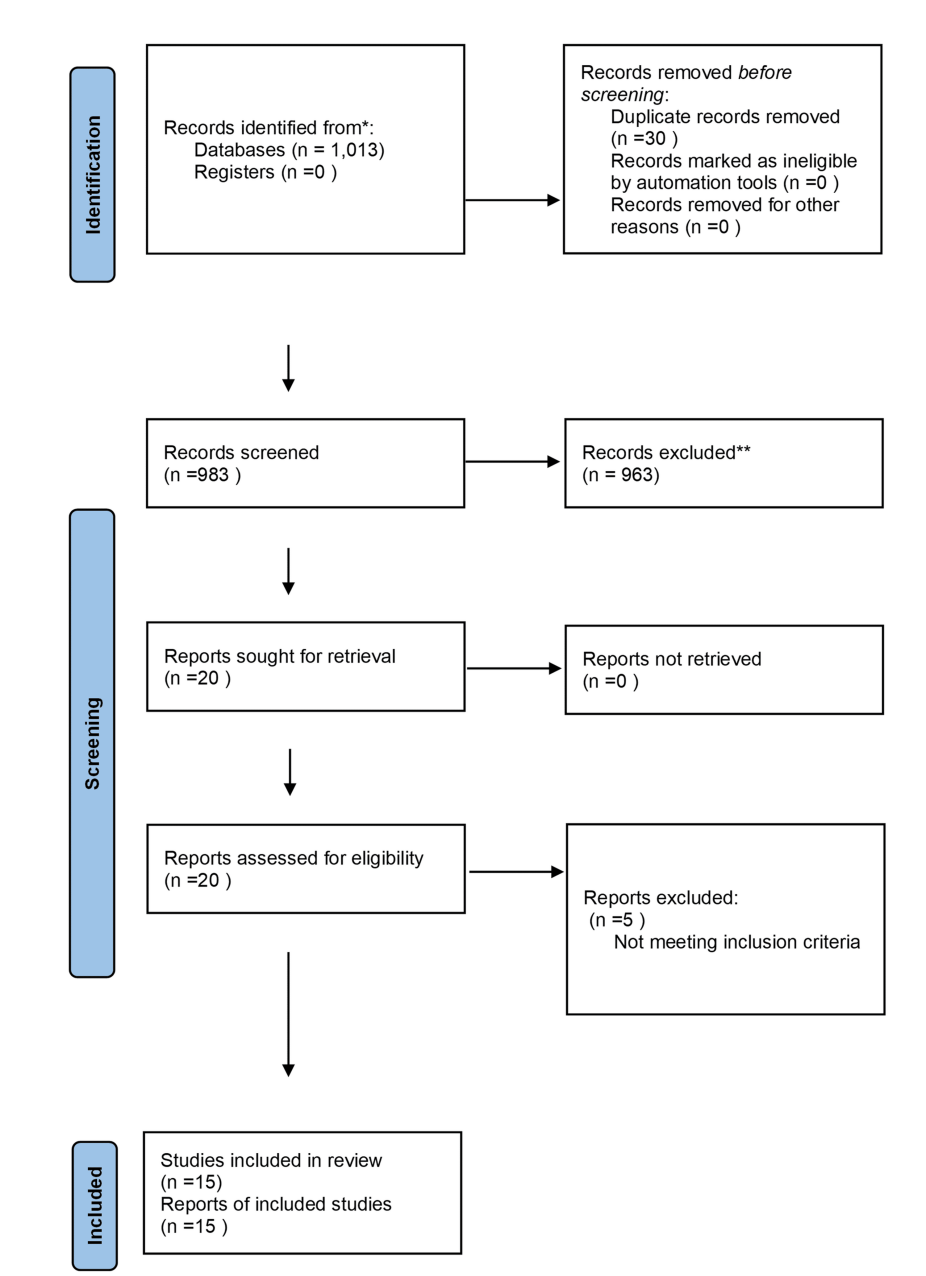
PRISMA flow diagram illustrating the study selection process for studies comparing direct versus indirect bonding of fixed orthodontic retainers, adapted according to PRISMA guidelines Source: [6]

The database was searched, and a total of 1,013 records were initially obtained. After removing duplicates, 983 studies were left. Title and abstract screening were done, which resulted in the exclusion of 963. Following a full-text assessment based on predefined inclusion and exclusion criteria, 15 studies were decided to be included in the final analysis. Thirteen studies [4,11-22] were RCTs, and two were prospective cohort studies [23,24]. Figure 1 shows the PRISMA flowchart which outlines the study selection process. A total of 1,481 patients were included in this meta-analysis. All included studies assessed the failure rates of mandibular fixed retainers, while only four studies [11,17,18,24] reported on failure rates of maxillary retainers using both direct and indirect bonding techniques.

**Figure 2:**
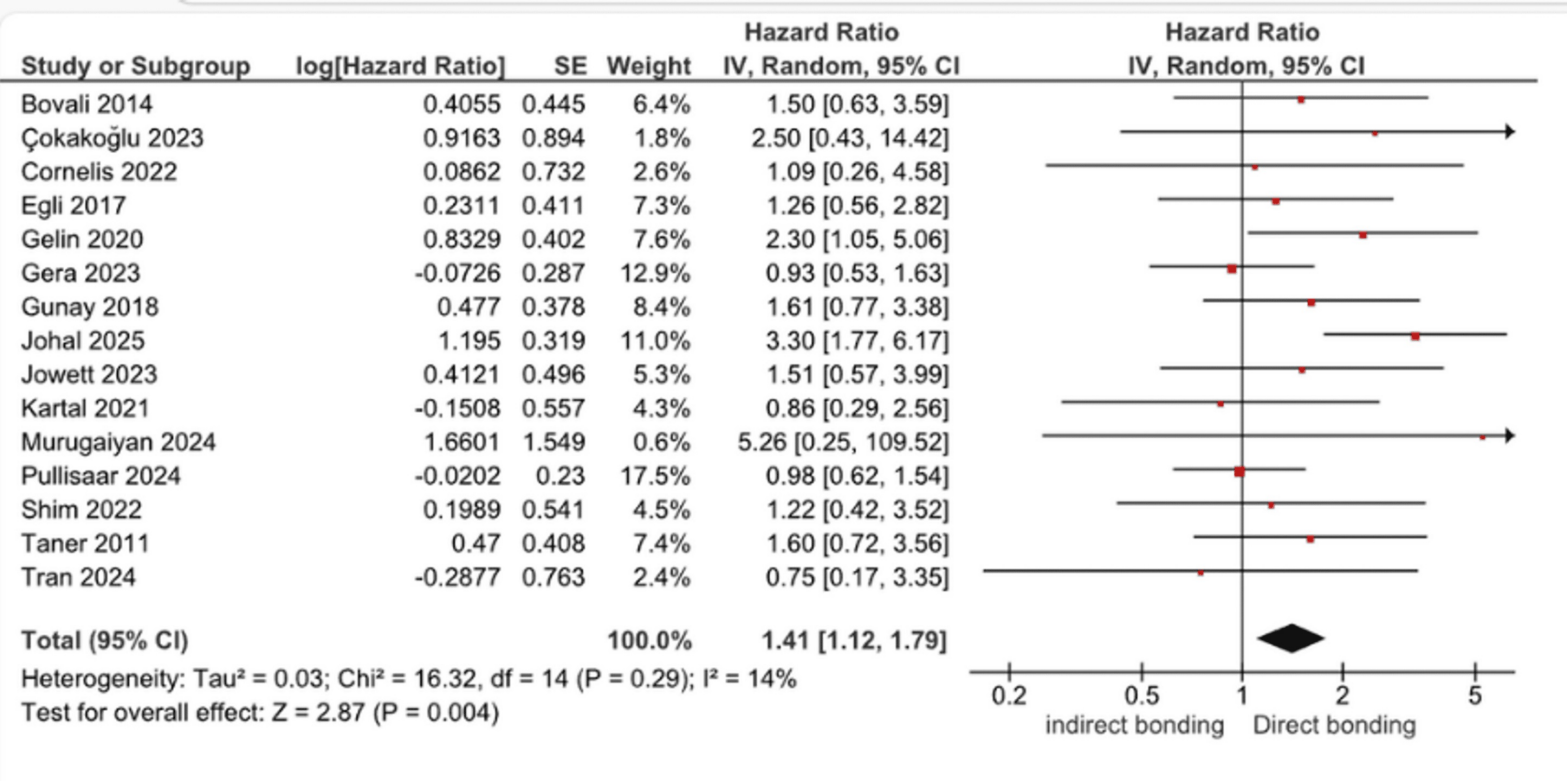
Forest plot for the lower arch This forest plot compares the failure risk of mandibular fixed retainers bonded using indirect versus direct techniques. Individual study hazard ratios were derived from included randomized and cohort studies [4,11–24].

A total of 15 studies compared the failure rates of indirect versus direct bonding for the mandibular arch (Table 1). The pooled analysis demonstrated a statistically significant difference between the two techniques, with a combined hR of 1.41 (95% CI: 1.12-1.79), p = 0.004. This indicates that directly bonded mandibular retainers have a higher risk of failure compared with those bonded indirectly. Figure 2 shows the forest plot of the included studies. Appendix B provides additional methodological detail supporting the summary of included studies.

**Table 1:** Characteristics of included studies comparing direct and indirect bonding techniques for fixed orthodontic retainers, including study design, sample size, follow-up duration, failure rates, hazard ratios, and statistical significance for mandibular and maxillary retainers. RCT: randomized controlled trial

Author/ study design	Retainer wire for direct bonding	Retainer wire for indirect bonding	Etchant for direct bonding	Etchant for indirect bonding	Adhesive for direct bonding	Adhesive for indirect bonding
Jowett et al. [11] / RCT	0.039 × 0.014-inch rectangular wire	Nickel-titanium (nitinol)	35% phosphoric acid	Same	Transbond XT Primer + Transbond LR	Same
Gera et al. [17] / RCT	0.0215-inch six-stranded stainless steel	0.014 × 0.014-inch rectangular nitinol	37% phosphoric acid	Same	Transbond XT + Tetric Flow	Same
Bovali et al. [4] / RCT	0.0215-inch multistrand stainless steel wire	Same	35% phosphoric acid	35% phosphoric acid	Transbond XT Primer + Transbond LR	Maximum Cure A & B
Gelin et al [20] / RCT	0.0175-inch, 6-strand twisted stainless steel	(Memotain®) 0.014 × 0.014-inch	37% phosphoric acid	Same	Transbond XT + Transbond LR	Same
Egli et al. [15] / RCT	0.0215-inch multistranded stainless steel	Same	Not reported	Not reported	Transbond XT + Transbond LR	Maximum Cure A & B (Reliance)
Gunay and Oz [12] / RCT	0.0195-in dead-soft coaxial wire	0.0175-in 6-strand multistranded stainless steel wire	32% phosphoric acid	Same	Transbond XT + Transbond LR	Same
Pullisaar et al. [18] / RCT	0.0215-inch 6-stranded stainless steel	0.014 × 0.014-inch nitinol (nickel-titanium)	Not specified (likely phosphoric acid)	Same	Not specified (likely standard protocol)	Same
Shim et al. [13] / RCT	Multistranded stainless steel	Same	35% phosphoric acid	Same	Assure Plus + FlowTain	Same
Cornelis et al. [16] / RCT	0.0215-inch multistranded stainless steel wire	Same	Not reported	Not reported	Transbond LR	Maximum Cure A & B (Reliance)
Çokakoğlu S, Kızıldağ [19] / RCT	0.0215" 5-strand stainless steel	Same	Not reported	Not reported	Transbond LR / Ortho Connect Flow	Maximum Cure / Ortho Connect Flow
Murugaiyan et al. [24] / prospective	0.0155-inch (0.40 mm) 6-stranded stainless steel	0.5 × 0.3 mm cobalt-chromium CAD	37% phosphoric acid	Same	Ortho Solo + NexcompFlow	Same
Taner and Aksu [23] / prospective	0.016 × 0.022 inch	Same	37% phosphoric acid	37% phosphoric acid	Transbond XT + LR	Maximum Cure A & B
Kartal et al. [14] / RCT	0.0215 in 5-strand stainless steel coaxial	0.014 × 0.014 in nickel–titanium	37% phosphoric acid	Same	Transbond XT Primer + LR	Same
Tran et al. [21] / RCT	Ortho-FlexTech (0.974 × 0.402 mm)	3-strand stainless steel wire (0.5 mm	35% phosphoric acid	Same	Assure Plus + FlowTain	Same
Johal et al. [22] / RCT	0.0383-in by 0.0158-in Ortho FlexTech retainer wire AND 0.025-in Blu-Elgiloy	0.0215-in multistranded gold-plated	37% phosphoric acid etching	Same	Transbond XT primer	Same

Individual study estimates varied, with some reporting higher failure rates for direct bonding (e.g., Murugaiyan et al. (2024) [24], HR: 5.26 (0.25-109.52)), while others favored indirect bonding (e.g., Kartal et al. (2021) [14], HR: 0.86 (0.29-2.56)). Despite this variability, heterogeneity across the studies was low (chi-square = 16.32, df = 14, P = 0.29; I² = 14%), indicating good consistency in the overall results.

A total of four studies [9,15,16,21] evaluated the failure risk of indirect (CAD/CAM) versus direct (conventional) retainers in the maxillary arch. Using a random-effects model, the pooled analysis showed no statistically significant difference between the techniques, with a combined HR of 1.30 (95% CI: 0.89-1.90), p = 0.18.

**Figure 3:**
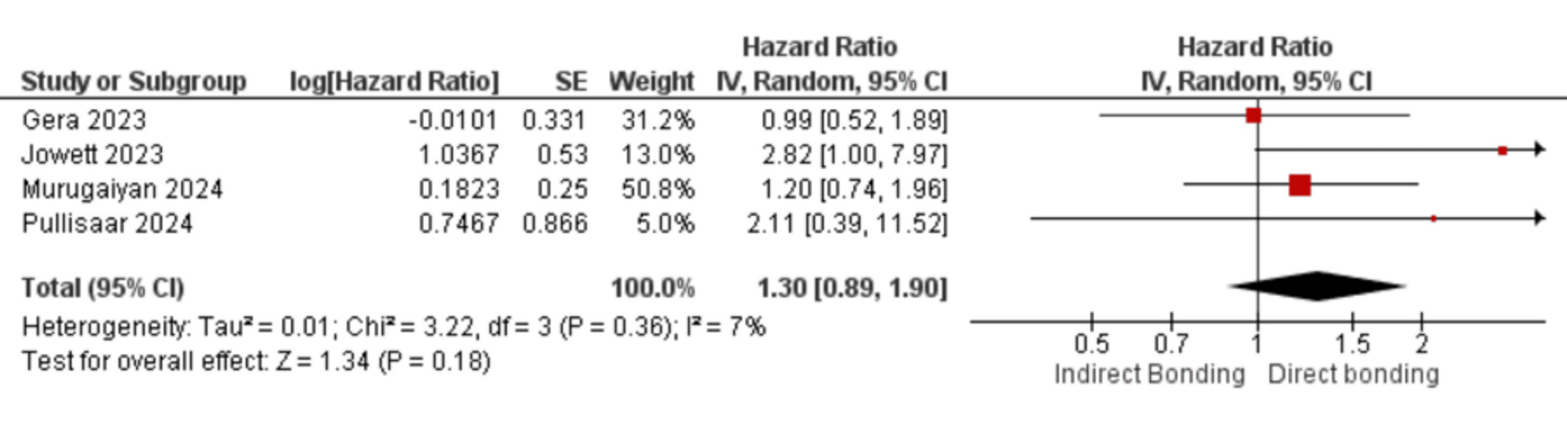
Forest plot for the upper arch This forest plot compares the failure risk of maxillary fixed retainers bonded using indirect versus direct techniques, based on included clinical studies [11,17,18,24].

Heterogeneity among these maxillary studies was low (chi² = 3.22, df = 3, P = 0.36; I² = 7%), suggesting consistent findings across the included trials. Figure 3 displays the forest plot for maxillary retainers.

Results of Individual Studies

In this meta-analysis, the retainer failure was assessed using failure rates and HRs. For each included study, we extracted the reported HR and its 95% CI whenever available.

Four studies [11,15,17,18] reported HRs directly, while 11 [4,12-14,16,19-24] studies provided only binary outcome data at a fixed follow-up and required HR estimation. 

Jowett et al.'s study (2023) [11] was terminated early due to a high failure rate in the indirect group (Memotain®). The failure rate for the indirect retainer of the upper (Memotain®) was 50%, and there was a 17% failure rate with the direct (Ortho-FlexTech™), and this was statistically significant. The HR was 2.82 (95% CI: 1.00-7.99; P = 0.019). The direct group (Memotain®) failure rate showed that there was a significantly increased risk of failure in the maxillary arch. In the mandibular arch, failure rates were 35% in the indirect group (Memotain®) and 28% for the direct group (Ortho-FlexTech™), but the difference was not statistically significant (HR = 1.51, 95% CI: 0.57-3.99; P = 0.53).

Gera et al.'s (2023) [17] failure rate was 22% of maxillary retainers for both direct and indirect groups, and approximately 30% of lower retainers for the indirect group and 29% for direct. However, this difference in the failure rate was not statistically significant in either the upper or lower arch. The reported HRs indicated that there is no statistically significant difference in survival between the two groups: the HR for the upper arch was 0.99 (95% CI: 0.52-1.90; P = 0.99), and for the lower arch it was 0.93 (95% CI: 0.53-1.63; P = 0.80).

In the study by Bovali et al. (2014) [4], the failure occurred in 32% of the indirect group and 24% of the direct group. This difference in the failure rate between the two groups was not statistically significant (P = 0.35). HRs were not reported in this study. We approximated the HR using the log odds ratio (OR) method; the estimated HR was 1.50 (95% CI: 0.48-4.68), indicating that there is no statistically significant difference in failure risk between the two groups.

In Gelin et al.'s study (2020) [20], the failure rate was 52.6% for the indirect group (CAD/CAM) and 40.9% for the direct group. Although HRs were not reported, we calculated an approximate HR using the log OR method; the estimated HR was 1.61 (95% CI: 0.47-5.54), indicating that there is no statistically significant difference in failure risk between the two groups.

In the study by Egli et al. (2017) [15], the failure rate was 43% for the indirect group and 37% for the direct group. And this difference was not statistically significant (P = 0.59). The HR was reported as 1.26 (95% CI: 0.56-2.81; P = 0.58), indicating that there is no significant difference in failure risk between the bonding techniques.

In the study by Gunay and Ozv (2018) [12], the failure rate was 13.2% in the stainless steel group and 18.9% in the dead-soft group, with no statistically significant difference between the two groups (P = 0.597). Although HRs were not reported, we calculated an approximate HR using the log OR method; the estimated HR was 1.43 (95% CI: 0.54-3.75), indicating that there is no statistically significant difference in failure risk between the two groups. Both groups used direct bonding; the stainless steel wire was fabricated on a study model and transferred to the mouth using a silicon key, so it’s considered semi-direct, while the dead-soft wire was shaped and bonded directly in the mouth, which may confound the interpretation of wire performance.

In the study by Pullisaar et al. (2024) [18], in the maxillary arch, the failure rate was 34% for the indirect group (CAD/CAM) and 38% for the direct group (conventional retainers). In the mandibular arch, the failure rate was 42% for the indirect group (CAD/CAM) and 40% for the conventional direct group. This difference was not statistically significant. The reported HR = 1.20 (95% CI: 0.74-1.95; P = 0.46) in the maxillary arch and HR = 0.98 (95% CI: 0.62-1.56; P = 0.94) in the mandibular arch, indicating that there is no significant difference in failure risk between the two groups.

Shim et al.'s study (2022) [13] was a three-arm RCT. The failure rates were 25% for CAD/CAM, 43.8% for lab-based, and 14.3% for traditional retainers. For meta-analysis purposes, the two conventional groups were combined, so the failure rate was 30%. The HRs were not reported, so we calculated an approximate HR using the log odds ratio method. The estimated HR was 2.00 (95% CI: 0.31-13.05), indicating that there is no statistically significant difference in failure risk between the two groups.

As reported by Cornelis et al. (2022) [16], the failure rate was 54% in both the direct and indirect groups. The authors reported no statistically significant difference in survival between the groups, with an HR of 1.09 (95% CI: 0.26-4.60; P = 0.91).

In the study by Çokakoğlu and Kızıldağ et al. (2023) [19], the failure rate was 9.1% in the direct group with conventional adhesive and 20% in the indirect bonding group with traditional adhesive. The HRs were not reported; we calculated an approximate HR using the log OR method. The estimated HR was 2.50 (95% CI: 0.43-14.40), indicating no statistically significant difference in failure risk between the two groups.

In the study by Murugaiyan et al. [24] (2024), in the mandibular arch, the failure rate was 20% for the direct group (bonded retainers) and 0% for the indirect (rapid prototype retainers). This difference was not statistically significant (P = 0.38). The HRs were not reported; we calculated them using the log OR method with continuity correction. The estimated HR was 6.88 (95% CI: 0.29-164.8), indicating that there is no statistically significant difference in failure risk between the two groups, and this is due to the wide CI.

In Taner and Aksu et al.'s study (2011) [23], the failure rate was 46.9% for the direct group and 29.4% for the indirect group. This difference in the failure rate was not statistically significant (P > 0.05). As the HR was not reported, we calculated the HR using the log OR method; the result was 2.11 (95% CI: 0.77-5.79), indicating that there is no significant difference in failure risk between the two groups.

In Kartal et al.'s study (2021) [14], the failure rate was 23% for the indirect (Memotain®) group and 27% for the direct (five-stranded) group. This difference was not statistically significant (P = 0.749). The HRs were not reported, so we calculated them using the log OR method. The estimated HR was 0.82 (95% CI: 0.23-2.89), indicating that there is no significant difference in failure risk between the two groups.

In Tran et al.'s study (2024) [21], the CAD/CAM and lab-based groups both used indirect bonding and were combined for this analysis. The failure rate in the indirect bonding group was 27.6% and 28.6% in the direct bonding (chairside) group. This difference in failure rates was not statistically significant (P = 0.14). An approximate HR was calculated using the log OR method; the calculated HR was 1.05 (95% CI: 0.25-4.35), indicating that there is no significant difference in failure risk between indirect and direct bonding techniques.

In the study by Johal et al. (2025) [22], the failure rate was 4% for the direct (Ortho-FlexTech™) group and 19.5% for the indirect groups (multistranded + Blu-Elgiloy). This difference was statistically significant (overall P < 0.001). The HRs were not reported for a direct-indirect comparison, so we calculated them using a pooled weighted method. The estimated HR was 0.14, indicating a markedly lower failure risk with direct bonding, although the statistical significance of this pooled estimate cannot be confirmed.

Risk of Bias Results

**Figure 4:**
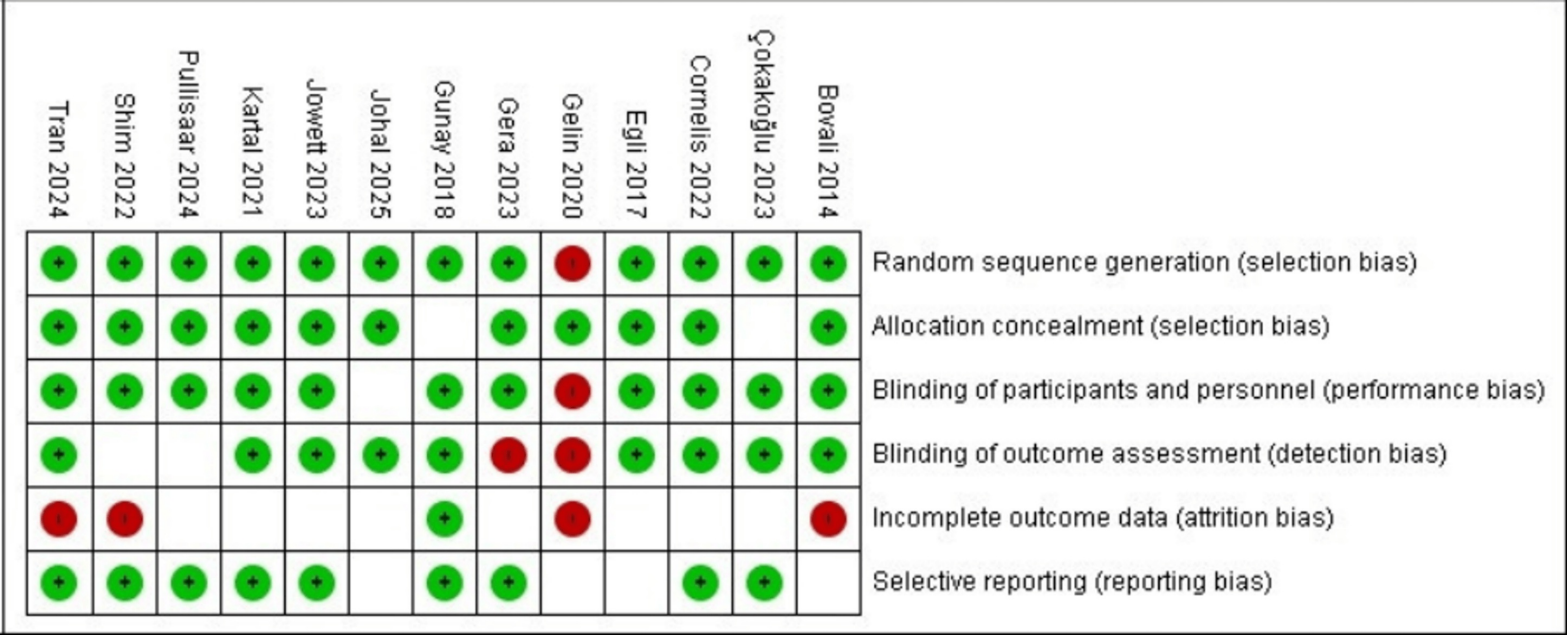
Risk-of-bias summary for included randomized controlled trials, assessed using the the Revised Cochrane Risk of Bias tool for randomized trials (RoB 2) Source: [8]

Overall, the included RCTs demonstrated low to moderate risk of bias across most domains (Figure 4), indicating generally acceptable methodological quality. The risk of bias related to missing outcome data and selective reporting was low in the majority of studies, while limitations were primarily related to blinding procedures.

Random sequence generation was judged to be at low risk in 12 studies, with one study assessed as high risk, suggesting a potential limitation in its randomization process. Allocation concealment was rated as low risk in 11 studies, while two studies [12,19] provided insufficient information, resulting in an unclear judgment. Blinding of participants and personnel (performance bias) and blinding of outcome assessment (detection bias) showed greater variability: 11 studies were at low risk for performance bias, and nine studies were at low risk for detection bias. A small number of trials, including ones by Bovali et al. (2014) [4], Gelin et al (2020) [20], and Shim et al. (2022) [13], were judged to have a high risk of bias in these domains due to the absence of blinding.

Incomplete outcome data were considered high risk in four studies, whereas the remaining trials were rated as unclear because of limited reporting on follow-up completeness or attrition. Selective reporting bias was assessed as low risk in nine studies, with the remainder judged as unclear owing to insufficient information on pre-specified outcomes or reporting transparency.

For the cohort studies, Murugaiyan et al. (2024) [24] demonstrated a low risk of bias, while Taner and Aksu (2011) [23] were judged to have a moderate risk of bias, primarily due to non-randomized allocation and limited control for confounding factors (Table 3). Cohort studies were evaluated separately from RCTs.

**Table 2:** Quality assessment of cohort studies using the Newcastle-Ottawa scale

Study	Selection (max 4)	Comparability (max 2)	Outcome (max 3)	Total (max 9)	Risk of bias
Murugaiyan et al. [24]	★★★★	★★	★★★	9	Low
Taner & Aksu [23]	★★★	★	★★	6	Moderate

Discussion

The meta-analysis included 15 mandibular datasets and demonstrated a statistically significant difference between bonding techniques. Indirect bonding showed superior survival, with a pooled HR of 1.41 (95% CI: 1.12-1.79; p = 0.004). Heterogeneity was low (I² = 14%), indicating that the direction and magnitude of the effect were consistent across studies despite variations in follow-up duration, wire type, and adhesive protocols. [25]

These findings suggest that indirect bonding-whether laboratory-prepared or CAD/CAM-assisted-may provide more reliable outcomes for mandibular fixed retainers. Improved pad adaptation, more controlled composite placement, and better isolation during bonding may contribute to this advantage, and for maxillary retainers, the HR was 1.30 (95% CI: 0.89-1.90), p = 0.18). Statistical heterogeneity was low (I² = 14% mandibular; 7% maxillary), supporting the consistency of findings across studies.

These results are consistent with multiple studies reporting no significant difference in failure rates between bonding techniques. For example, Gera et al. (2023) [17] and Gelin et al. (2020) [20] found similar survival outcomes regardless of the technique used. Notably, Jowett et al. (2023) [11] identified a significantly higher failure rate for indirectly bonded Memotain® retainers in the maxillary arch, though such findings were not replicated consistently across the broader dataset. This variability highlights that technique alone may not be the primary determinant of retainer longevity.

Several factors may explain the comparable outcomes observed. Both direct and indirect bonding methods rely heavily on operator skills, material properties, and patient compliance [26]. Although indirect bonding theoretically offers greater precision and reduced chair time, the additional laboratory steps introduce opportunities for technical errors. Conversely, direct bonding, while potentially more prone to in-situ variability, allows real-time adjustments by the clinician.

Importantly, not all indirect bonding techniques in the included studies were CAD/CAM-based, and some CAD/CAM retainers were bonded directly. Therefore, caution should be exercised when interpreting indirect bonding as synonymous with CAD/CAM technology. Future subgroup analyses focused on CAD/CAM versus traditional wire retainers may yield more granular insights into material performance.

From a clinical standpoint, the demonstrated advantage of indirect bonding in the mandible, coupled with comparable outcomes in the maxilla, suggests that both techniques remain acceptable options. Thus, technique selection may reasonably depend on operator preference, workflow, and patient-specific factors instead of being dictated solely by failure rates

However, this meta-analysis has some limitations. First, although most included studies were RCTs with low to moderate risk of bias, blinding of outcome assessment was inconsistently applied. Second, the definition of "failure" varied slightly across studies, and not all studies reported HRs directly; 10 of 15 studies required estimation from binary outcomes data [10]. Third, variations in retainer wire types, adhesives, and follow-up durations introduce some heterogeneity that could not be entirely controlled.

Future research should focus on standardising definitions of retainer failure and ensuring uniform follow-up periods. Additionally, more high-quality RCTs directly comparing different CAD/CAM retainer systems with conventional methods would be valuable, particularly assessing long-term outcomes beyond 24 months.

## Conclusions

This meta-analysis demonstrates a significant advantage of indirect bonding for mandibular fixed retainers, with a 41% relative reduction in failure risk compared with direct bonding. In contrast, no significant difference between bonding techniques was observed for maxillary retainers.

Indirect bonding-whether conventional laboratory-based or CAD/CAM-assisted-may therefore offer improved reliability for mandibular retainer placement. Nonetheless, further high-quality, long-term RCTs are needed to confirm these findings and to clarify the performance of different CAD/CAM systems.

## Appendices

Appendix A

(Dental[tiab] OR Dentist*[tiab] OR Orthodont*[tiab] OR orthodontic retention[tiab] OR orthodontic retainer[tiab])

AND (Mandibular fixed retainers[tiab] OR mandibular retainers[tiab] OR direct bonded retainer[tiab]

OR indirect bonded retainer[tiab] OR mandibular lingual retainer[tiab] OR "Orthodontic Retainers"[Mesh]

OR retainer failure*[tiab] OR lingual retainer survival[tiab] OR retainer bonding technique[tiab]

OR retainer survival[tiab] OR bonding technique [tiab])

Appendix B

**Table 3:** Summary of retainer wire types, etching protocols, and adhesive systems used in the direct and indirect bonding groups across the included studies.

Author /study design	Sample (n)	Follow-up (m)	Failure rate for the indirect lower arch	Failure rate for the indirect upper arch	Failure rate for the direct lower arch	Failure rate for the direct upper arch	Hazard ratio (HR) for the lower arch	Hazard ratio (HR) for the upper arch	HR reported or calculated	P-value for lower	Significant or non-significant	P-value for the upper arch	Significant or non-significant
Jowett et al. [11] / RCT	68	6 m	35%	50%	28%	17%	1.51	2.82	Reported	0.53	NS	0.019 for upper	SS
Gera et al. [17]. / RCT	181	12m	30%	22%	29%	22%	0.93	0.99	Reported	0.8	NS	0.99 for upper	NS
Bovali et al. [4] / RCT	64	6m	32.2%	Not reported	24.1%	Not reported	1.5	Not reported	Calculated	0.35	NS	Not reported	Not reported
Gelin et al. [20] / RCT	62	12m	32.3%	Not reported	29%	Not reported	1.11	Not reported	Calculated	Not reported	NS, the author states that it is not statistically significant	Not reported	Not reported
Egli et al. [15] / RCT	64	24 m	43.3%	Not reported	36.7 %	Not reported	1.26	Not reported	Reported	0.58	NS	Not reported	Not reported
Gunay and Oz [12] / RCT	120	12m	13.2 %	Not reported	18.9 %	Not reported	1.43	Not reported	Calculated	0.597	NS	Not reported	Not reported
Pullisaar et al. [18] / RCT	181	24m	42 %	34%	40 %	38%	0.98	1.2	Reported	0.94	NS	0.46 for upper	NS
Shim et al. [13] / RCT	75	6m	20%	Not reported	16.36 %	Not reported	1.22	Not reported	Calculated	0.045	S	Not reported	Not reported
Cornelis et al. [16] / RCT	64	60 m	54%	Not reported	54%	Not reported	1.09	Not reported	Calculated	0.91	NS	Not reported	Not reported
Çokakoğlu and Kızıldağ [19] / RCT	100	12 m	22%	Not reported	54%	Not reported	2.5	Not reported	Calculated	0.459	NS	Not reported	Not reported
Murugaiyan et al. [24] / Prospective	41	12 m	0%	9.5%	0 %	20%	5.26	2.11	Calculated	0.43	NS	0.38	NS
Taner et al. [23] / Prospective	66	6 m	29.4 %	Not reported	46.9%	Not reported	1.6	Not reported	Calculated	Not reported	NS	Not reported	Not reported
Kartal et al. [14] / RCT	52	6 m	23%	Not reported	27%	Not reported	0.86	Not reported	Calculated	0.749,	NS	Not reported	Not reported
Tran et al. [22] / RCT	43	24 m	28.8 %	Not reported	27.6%	Not reported	1.05	Not reported	Calculated	Not reported	NS	Not reported	Not reported
Johal et al. [22] / RCT	300	24 m	19.5%	Not reported	4%	Not reported	0.14	Not reported	Calculated	Not reported	S	Not reported	Not reported
